# Classification of Amyloidosis by Model-Assisted Mass Spectrometry-Based Proteomics

**DOI:** 10.3390/ijms23010319

**Published:** 2021-12-28

**Authors:** Nicolai Bjødstrup Palstrøm, Aleksandra M. Rojek, Hanne E. H. Møller, Charlotte Toftmann Hansen, Rune Matthiesen, Lars Melholt Rasmussen, Niels Abildgaard, Hans Christian Beck

**Affiliations:** 1 Odense Amyloidosis Center, Odense University Hospital, 5000 Odense, Denmark; Nicolai.Bjodstrup.Palstrom@rsyd.dk (N.B.P.); aleksandra.maria.rojek@rsyd.dk (A.M.R.); hanne.moeller@rsyd.dk (H.E.H.M.); charlotte.toftmann.hansen2@rsyd.dk (C.T.H.); Lars.Melholt.Rasmussen@rsyd.dk (L.M.R.); niels.abildgaard@rsyd.dk (N.A.); 2 Centre for Clinical Proteomics, Department of Clinical Biochemistry and Pharmacology, Odense University Hospital, 5000 Odense, Denmark; 3 Department of Pathology, Odense University Hospital, 5000 Odense, Denmark; 4 Department of Hematology, Odense University Hospital, 5000 Odense, Denmark; 5 Computational and Experimental Biology Group, CEDOC, Chronic Diseases Research Centre, NOVA Medical School, Faculdade de Ciências Médicas, Universidade NOVA de Lisboa, 1169-056 Lisbon, Portugal; rune.matthiesen@nms.unl.pt; 6 Department of Clinical Research, Odense University Hospital, 5000 Odense, Denmark; 7 Haematology Pathology Research Laboratory, Department of Haematology, Odense University Hospital, 5000 Odense, Denmark

**Keywords:** amyloidosis, mass spectrometry, laser microdissection, proteomics, machine learning

## Abstract

Amyloidosis is a rare disease caused by the misfolding and extracellular aggregation of proteins as insoluble fibrillary deposits localized either in specific organs or systemically throughout the body. The organ targeted and the disease progression and outcome is highly dependent on the specific fibril-forming protein, and its accurate identification is essential to the choice of treatment. Mass spectrometry-based proteomics has become the method of choice for the identification of the amyloidogenic protein. Regrettably, this identification relies on manual and subjective interpretation of mass spectrometry data by an expert, which is undesirable and may bias diagnosis. To circumvent this, we developed a statistical model-assisted method for the unbiased identification of amyloid-containing biopsies and amyloidosis subtyping. Based on data from mass spectrometric analysis of amyloid-containing biopsies and corresponding controls. A Boruta method applied on a random forest classifier was applied to proteomics data obtained from the mass spectrometric analysis of 75 laser dissected Congo Red positive amyloid-containing biopsies and 78 Congo Red negative biopsies to identify novel “amyloid signature” proteins that included clusterin, fibulin-1, vitronectin complement component C9 and also three collagen proteins, as well as the well-known amyloid signature proteins apolipoprotein E, apolipoprotein A4, and serum amyloid P. A SVM learning algorithm were trained on the mass spectrometry data from the analysis of the 75 amyloid-containing biopsies and 78 amyloid-negative control biopsies. The trained algorithm performed superior in the discrimination of amyloid-containing biopsies from controls, with an accuracy of 1.0 when applied to a blinded mass spectrometry validation data set of 103 prospectively collected amyloid-containing biopsies. Moreover, our method successfully classified amyloidosis patients according to the subtype in 102 out of 103 blinded cases. Collectively, our model-assisted approach identified novel amyloid-associated proteins and demonstrated the use of mass spectrometry-based data in clinical diagnostics of disease by the unbiased and reliable model-assisted classification of amyloid deposits and of the specific amyloid subtype.

## 1. Introduction

Amyloidosis is a term used to describe a group of rare and serious diseases that are characterized by deposition of abnormal proteins in a characteristic fibrillary form in the extracellular matrix of various vital tissues and organs. Presently, more than 35 proteins have been shown to form amyloid deposits in humans [1]. The specific amyloidogenic protein determines the sub-classification of the amyloidosis with immunoglobulin light chain (AL), transthyretin (ATTR), and serum amyloid A (AA) as the most important and frequent ones.

Where the symptoms and the clinical presentation of amyloidosis may be very different and organ or tissue-dependent, they are almost identical across amyloid subtypes [2,3]. Thus, as treatment—spanning from chemotherapy for AL amyloidosis to organ transplantation of the liver or heart for ATTR-related amyloidosis—and the prognosis are radically different for each of the individual amyloid subtypes, precise and accurate diagnostic sub-classification of the amyloid fibrillary protein in each identified subject is of outmost importance for selection of treatment regime.

Traditionally, sub-type determination of the amyloidogenic protein was based on immune-histochemical analysis of biopsies from the affected organ or tissue [4,5]. This method has, however, been discarded in many clinical pathology departments due to low sensitivity and low specificity [5,6,7,8], the latter presumably caused by unspecific staining. Although, some diagnostic laboratories routinely achieve a sensitivity higher than 90% using immunohistochemistry (IHC) [9]. Recently, other methods for identifying the amyloid fibril proteins include laser microdissection (LMD) of amyloid deposits visualized by Congo Red (CR) staining combined with mass spectrometry (MS) or immune electron microscopy (IEM) [10,11] for the classification of localized amyloidosis and mass spectrometry-based shotgun proteome analyses of fat biopsies for the classification of systemic amyloidosis [12]. A major advantage with mass spectrometry-based methods for amyloidosis subtyping is that this method not only measures the amyloidogenic protein in question but also measures an amyloid protein signature that is shared across all amyloidosis subtypes in various tissues in a highly specific and quantitative manner. Common for these methods is, however, that evaluation of the amyloid protein signature characteristics for amyloid plaques, and the identification of the subtype-specific protein in question relies on manual inspection of MS-data and is, therefore, highly reliant on the person interpreting the results from the analysis. In clear-cut cases with severe levels of the amyloidogenic protein, this is not of concern. Oftentimes, however, the MS-data are ambiguous, and a certain degree of subjective interpretation by an MS expert is required. This subjective interpretation is undesirable as it may bias the result leading to the wrong subtype diagnosis. 

In the present work, we hypothesized that subjective interpretation can be avoided by developing classification models for the subtyping of amyloidosis by training machine learning algorithms on mass spectrometry proteomics data. Classification models were trained and tested on proteomics data from the analysis of amyloid-containing biopsies with known amyloidogenic proteins and validated by the application of the developed classification models on proteomics data from the analysis of 103 blinded amyloid-containing laser dissected biopsies.

## 2. Results

The 75 CR-positive amyloid-containing samples and the 78 CR-negative samples without amyloid were measured by nano-LC-MSMS which identified 1862 proteins across all samples. Spectral counts (i.e., PSMs) for each of the identified proteins served as quantitative measures (Supplementary Table S2). Quantitative data from the proteomic analysis of the 153 biopsies were divided into a training set (70% of the 153 samples) and a test set (30% of the 153 samples) randomly chosen among the 75 CR-positive and 78 CR-negative samples. A Boruta feature selection method applied to a random forest classifier was applied to estimate the capacity of each of the 1862 identified proteins of the merged dataset to differentiate between the CR-positive and CR-negative samples. Among the 10 proteins with the highest capacity to differentiate between CR-positive and CR-negative samples, we identified ApoA4, ApoE, SAP and Complement component C9, as well as Clusterin and Vitronectin (Table 1). Moreover, three collagen chains, as well as fibulin-1, also demonstrated a high capacity to discriminate amyloid-containing tissue biopsies from non-amyloid tissue samples.

Next, we evaluated the capability of the identified amyloidosis signature proteins shown in Table 1—either alone or in combinations—to identify CR-positive amyloid-containing biopsies from and CR-negative biopsies by using a model based on the SVM algorithm (Table 2 and Supplementary Table S3). Classification models were developed by training the algorithms on proteomics data from each of the 10 proteins shown to be associated with amyloid deposits (Table 1) using proteomics data from the analysis of the training set samples. The developed models were then tested on proteomics data from the test dataset. Clearly, each of the proteins ApoA4, ApoE, SAP and Clusterin demonstrated a great ability to discriminate between CR-positive and CR-negative samples displaying overall accuracies > 0.96 (Table 2), whereas combinations of the well-known amyloid-associated proteins and novel amyloid-associated proteins demonstrated overall accuracies between 0.93 and 1.00. Moreover, the combination of ApoA4, ApoE and Clusterin appeared with a similar performance as the well-established amyloid-signature (ApoA4, ApoE and SAP). 

A validation dataset consisting of 103 tissue samples with amyloid deposits prospectively collected from various organs was applied to validate the SVM-based models. (Table 3). The models based on either ApoA4 or ApoE alone performed equally well by classifying 99 out of 103 correctly. By contrast, the model based on SAP alone accurately classified all samples correctly as amyloid-containing tissue samples. The model based on Complement component C9 performed considerably worse than the models for remaining proteins by classifying only 26 tissue samples out of 103 biopsies correctly. The model based on ApoA4 and ApoE only marginally improved with the inclusion of each of the eight other identified signature proteins except for the model for the combination of ApoA4 and ApoE with SAP that identified all amyloid-containing tissue samples.

### Model-Assisted Typing of Amyloidosis Based on Proteomics Data

An SVM-based model for subtyping of amyloid-containing tissue biopsies (CR-positive laser dissected samples was trained on 54 randomly chosen amyloid-containing samples (70% of 75 the amyloid-containing tissue samples with known amyloidogenic proteins)). Quantitative measures (based on spectral counting) from seven proteins that have the capacity to differentiate between the four amyloidosis subtypes, AA, ATTR, AL-kappa, and AL-lambda light chain amyloidosis, were selected (Table 4). 

When testing our model on the training data set and the test data set it demonstrated a cross-validation accuracy of 1.00, i.e., all samples were correctly subtyped (results not shown). When applying our model to the subtyping of the 103 blinded samples from the validation dataset, the model using the four most prominent amyloidogenic proteins (four-protein model) correctly subtyped 99 samples misclassifying four ATTR cases as AL-lambda, whereas the seven-protein model correctly subtyped 102 samples demonstrating accuracy of >0.99. Only one sample diagnosed as ATTR by IEM was incorrectly annotated as AL-kappa by the seven-protein model (Table 5). 

## 3. Discussion

Amyloidosis is a disease, which requires skilled professionals to diagnose. The application of LMD and MS in the diagnosis of amyloidosis has greatly increased the efficiency of diagnosis and its application has gained a complementary role to IHC and IEM. A major drawback of mass spectrometry-based methods is, however, that the evaluation of the amyloid protein signature and the identification of the subtype-specific protein is highly reliant on the person interpreting the results of the analysis. In this study, we hypothesized that the application of a machine learning-based algorithm for recognition of the distinct patterns associated with amyloidosis could provide the MS expert with an unbiased tool that enables the accurate diagnosis of amyloidosis. The need for an unbiased tool for subtyping of systemic amyloidosis based on MS analysis of subcutaneous adipose tissue was previously addressed by Brambilla et al. [12] and Canetti et al. [13]. They developed an algorithm for the calculation of a diagnostic α-value for the subtyping of amyloidosis based on MS data and a simple empirical algorithm based on Mascot scores, respectively. The α-value aiming at systemic amyloidosis subtyping of the four most commonly occurring amyloidosis subtypes (AL-Lambda, AL-Kappa, ATTR, and SAA) should be 70 or higher for correct diagnosis. The α-value is a normalized parameter that is calculated based on the measured spectral counts for each of the four amyloidogenic proteins from the analysis of the CR-positive fat aspirates and a number of CR-negative samples. We applied this method to our data set but found that it was not applicable to MS data from the analysis of laser-dissected samples, presumably due to sampling inhomogeneity of CR-negative control samples, both within and particular across tissue types (results not shown). A major drawback with this method is, however, that the calculation of the α-value relies on MS data from the analysis of a number of CR-negative biopsies along with the analysis of a CR-positive sample, which is not always readily available in routine diagnostics. We, therefore, aimed to develop an algorithm that enabled the classification of CR-positive cases. We applied a feature selection method to identify specific proteins in a data set comprised of quantitative readout for 1862 proteins resulting from the analysis of 153 laser-dissected CR-positive and CR-negative biopsies. Among the proteins that showed the highest mean important score (i.e., with the feature to identify CR-positive cases), we expectedly identified the well-known amyloid-deposit signature proteins, ApoAIV, ApoE, and SAP. Interestingly, our feature selection algorithm identified seven additional proteins that characterized amyloid-containing tissue biopsies. These included complement C9, Clusterin, COL6A1, COL6A2, COL6A3, Fibulin-1 and Vitronectin. Clusterin, known to interact and mediate the clearance of Aβ, and Vitronectin have both already been associated with various subtypes of amyloidosis [14]. By contrast, Complement C9 has previously been associated with Alzheimer’s disease [15], but all three proteins are associated with the soluble membrane attack complex (SC5b-9) [16,17], but their role in amyloidosis fibrillogenesis remain elusive. Recently, Lux and co-workers noticed that Complement C9 was a dominating protein in the MS analysis of CR-positive routine diagnostic samples. Based on this observation they systematically explored the presence of Complement C9 in 118 tissue samples from 18 different tissue types with amyloid deposits [18]. They found that the immune reactivity of Complement C9 covered more than 80% of the CR positive area in more than 90% of the cases across all biopsy types, which confirms the appearance of this protein in our statistical analysis. Collagen is known to be stained by CR and display birefringence that can be mistaken for amyloid plaques [19,20]. It is likely that collagen-rich areas of the biopsies are laser-dissected along with amyloid plaques as it is not possible to distinguish between these two types of fibrils during excision of fibrillary areas of the biopsies in the laser microscope. We tested the capability of the ten proteins with the highest mean importance score to differentiate between CR-positive and CR-negative samples individually or in combination with each other by building models based on SVM-based algorithms. All individual proteins demonstrated a relatively high capacity to identify CR-negative samples (specificities ranging from 0.86 to 1.0). By contrast, only ApoA4, ApoE, SAP and Clusterin demonstrated a high capacity to identify CR-positive samples (sensitivities ranging from 0.9 to 1.0). Application of models based on the amyloid signature proteins ApoE and ApoA4 either alone or in combination with each of the other proteins tested showed that addition of Clusterin, Vitronectin, Complement C9, or SAP increased the sensitivity of the model from 0.91 to 1.0, whereas the addition of Clusterin, Fibulin-1 or SAP to the model increased the specificity from 0.96 to 1.0. When applying the model on data from the proteomic analysis of the 103 CR-positive samples from the blinded validation dataset, only the models that were built on SAP alone, or SAP in combination with ApoA4 and ApoE were capable of identifying all 103 blinded samples as CR-positive samples, in all confirming APOE, SAP and APOA4 as excellent surrogate biochemical markers for the presence of amyloid [21]. Although Clusterin, Vitronectin, or Complement C9 slightly improved the classification of CR-negative and CR-positive samples their role in the formation of amyloid deposits remains unclear. The second step focused on creating a model for accurate subtyping of amyloid positive cases. The model showed a high degree of accuracy as only a single ATTR case was misclassified as AL-K among the 103 cases of the blinded validation data set. The misclassified case in question had similarly low levels of both AL-K and ATTR-related proteins, which demonstrates the difficulties in diagnosing patients with amyloidosis. Although machine learning-based algorithms applied to the diagnosis of amyloidosis are not new. Machine learning-based algorithms have previously been applied to identify heart failure-related cardiac amyloidosis patients from heart failure-unrelated cardiac amyloidosis patients by the utilization of basic laboratory methods [22].To the best of our knowledge, the current study is the first to apply machine-learning to proteomics-based data for subtype classification of amyloidosis. In this work, SVMs were employed as it is a robust learning algorithm for classification with high discriminative power and insensitive to outliers. Nonetheless, evaluation of the performance of each model on a test dataset limits the risk of over-fitting the model and the further validation on an independent blinded validation dataset ensures that the tendencies seen in the test data set are robust.

## 4. Materials and Methods

### 4.1. Clinical Specimens

Two independent sets of amyloidosis specimens of various tissue types received as a part of the routine clinical practice at the Amyloidosis Centre at Odense University Hospital, Odense, Denmark were studied. The first set comprised 75 amyloid-containing CR-positive sections from various tissues, and 78 Congo-negative areas dissected from Congo positive samples of various tissue origins (Supplementary Table S1). These specimens were in a recent study characterized by standard laser dissection microscopy mass spectrometry analysis and immune-electron microscopy [10], and only specimens with 100% concordance between the LMD-MS analysis and IEM analysis were included in the present study. The second set consisting of 103 Congo-positive amyloid-containing specimens from various tissue types prospectively analyzed the combination of LMD-MS and the developed classification models in a blinded fashion. The project was approved by the local Ethics Committee (J.nr. S-20180128) and registered at the Danish Data Protection Agency (J.nr. 18/54959). 

### 4.2. Immuno-Electron Microscopy and Mass Spectrometry

#### 4.2.1. Microdissection and Sample Processing

Preparation and processing of samples for IEM analysis and mass spectrometry analysis were performed as previously described [10]. Briefly, for IEM analysis, ultrathin (70 nm) sections stained with toluidine blue containing histological structures regions with potential amyloid deposits as identified by light microscopy were probed by the antibody of interest (anti-Serum amyloid A, anti-Kappa- and Lambda Light chains, and anti-Prealbumin (Transthyretin) followed by incubation with Protein A—10 nm gold conjugate and visualization of amyloidogenic fibrils by electron microscopy. Immunostaining quality was validated by examination of positive controls stained in parallel with the investigated samples. 

For LMD-MS analysis 8 µm thick sections of formalin-fixed, paraffin-embedded (FFPE) patient specimens were fixated onto membrane slides, de-paraffinized, and CR stained for visualization of amyloid deposits. From each CR-positive patient biopsy, CR-negative areas were collected and used as controls together with dissected areas from CR-negative patient biopsies. Dissected areas (the total area was approx. 0.2 mm^2^) were prepared for proteome analysis as described in the previous study. Briefly, samples were incubated in 35 µL 10 mM Tris with 1 mM EDTA and 0.002% Zwittergent at 98 °C for 90 min followed by reduction (50 mM DTT, 50 °C, 30 min) and alkylation (150 mM IAA, RT in the dark, 30 min). Proteins were then acetone precipitated and re-dissolved in 20 µL, 200 mM triethylammonium bicarbonate (TEAB) followed by overnight digestion with 0.1 µg trypsin at 37 °C. Purification of the resulting tryptic peptides were carried out using custom made C18 microcolumns and the eluate was vacuum-centrifuged to dryness (SpeedVac, Thermo Scientific, Waltham, MA, USA) and reconstituted in 0.1% TFA for analysis by nano-LC-MSMS, as described below. 

#### 4.2.2. Liquid Chromatography and Mass Spectrometry

LC-MSMS was performed on an UltiMate3000 UHPLC unit coupled online to a Q-Exactive mass spectrometer fitted with a nano-electrospray ion source. Samples were loaded onto a custom-made, fused capillary pre-column (2 cm length, 360 µm OD, 75 µm ID packed with ReproSil Pur C18 3 µm resin (Dr. Maish, GmbH)) with a flow of 0.3 µL/min for 7 min. Trapped peptides were subsequently separated with a custom-made fuse capillary column (20 cm length, 360 µm OD, 100 µm ID, packed with ReproSil Pur C18 3 µm resin) employing a linear gradient from a 95% solution A (0.1% FA) toward a 28% solution B (100% acetonitrile in 0.1% FA) over a 52-min interval with a subsequent 5-min interval at 90% B and 5-min 95% A, with a flow rate of 0.3 µL/min. The Orbitrap MS scan was set to a target value of 1,000,000 ions at a resolution of 70,000 at *m/z* 200 and the MS/MS scan was set to a target value of 50,000 ions at a resolution of 17,500 at *m/z* 200 (fixed first mass 110 *m/z*). Fragmentation occurred at a normalized collision energy of peptides in the HCD cell at 32 eV and the intensity threshold for data-dependent MSMS analysis was 27,000 counts/s. 

### 4.3. Data Analysis

All raw data files were processed using the Proteome Discoverer software (v. 2.4.0.305) and searched with the Sequest HT search algorithm. The search parameters were set to an MS accuracy of 8 ppm, MSMS accuracy of 0.05 Da for HCD data, with two missed cleavages allowed. Fixed modifications included carbamidomethylation at cysteine residues and variable modifications included methionine oxidation, deamidation of asparagine and glutamine and N-terminal acetylation. Raw data files were searched against the Swiss-Prot database restricted to the human proteome (downloaded on the 12th of December 2019, containing 20,303 entries). Proteins identified with at least one unique peptide and with a high confidence (FDR < 1%) were permitted in the final dataset. 

For all patient samples, the number of peptide spectrum matches (PSMs) for the amyloid signature proteins (ApoA4, ApoE, and SAP) and the proteins associated with the four subtypes included in this study (IG-K, IG-L, SAA, and TTR) were used to determine the true disease-state of a patient. The amyloid-associated protein with the highest number of PSMs was determined to be the pathogenic protein, a prerequisite that at least two out of the three amyloid signature proteins were also detectable in the patient sample. 

### 4.4. Statistical Analyses

A Support Vector Machine (SVM), which is a supervised machine learning technique, implemented in the public available R package e1071, was applied for the classification of disease-state (amyloid-containing tissue or not) and classification of amyloidosis subtype. All SVM classifiers are based on linear kernel functions and variables are by default scaled to zero mean and unit variance by the *svm* function, included in the e1071 package, prior to classifier training. Selecting the optimal parameters of C and gamma for each SVM classifier was done using the *tune.svm* function (e1071). Feature selection was performed primarily as a pre-processing step to eliminate redundant and noisy protein data and secondly to evaluate proteins that could differentiate between amyloidosis-positive and amyloidosis-negative patients. Feature selection was performed using the Boruta feature selection algorithm [DOI: 10.18637/jss.v036.i11]. All prediction classifiers were optimized on the training dataset and performance was evaluated on a separate test dataset, as well as validated on a blinded, independent validation dataset. Computation of sensitivity, specificity, positive prediction value (PPV), negative prediction value (NPV), as well as diagnostic accuracy was achieved using the *confusionMatrix* function in the caret R package. The work was carried out under R (v. 4.02) in RStudio (V. 1.2.5001). All the applied R codes and datasets are available to download from the GitHub repository associated with this study (https://git.io/JMIqS, accessed on 30 November 2021).

## 5. Conclusions

In this study, we aimed to improve the diagnosis of amyloidosis by developing unbiased models based on proteomics data for the recognition of amyloid-containing biopsies followed by the accurate subtyping of amyloidosis. We demonstrated that the utilization of machine learning on proteomics data can identify and classify patients with high accuracy on a blinded validation data set of more than a hundred patients. Future studies should focus on the implementation of classification models in mass spectrometry-based amyloidosis subtyping.

## Figures and Tables

**Table 1:** Signature proteins for amyloid deposits. The Boruta feature selection method was applied to estimate the capacity of specific proteins to differentiate between Congo Red-positive and negative samples determined by the calculated “mean importance score “. The quantitative readout from the proteomic analysis (number of peptide spectrum matches for each protein) of the 153 biopsies divided into a training set (70% of the biopsies randomly chosen among CR+ and CR− samples) and a test set (30% of the biopsies randomly chosen among CR+ and CR− samples).

Rank	#Accession	Protein Name	Mean Importance
1	P10909	Clusterin	10.94
2	P02743	Serum amyloid P-component	10.47
3	P06727	Apolipoprotein A-IV	9.99
4	P04004	Vitronectin	9.20
5	P02649	Apolipoprotein E	8.94
6	P02748	Complement component C9	7.25
7	P12109	Collagen alpha-1(VI) chain	6.25
8	P12110	Collagen alpha-2(VI) chain	5.19
9	P12111	Collagen alpha-3(VI) chain	4.93
10	P23142	Fibulin-1	4.83

**Table 2:** The capability of the identified amyloid signature proteins, and combinations of these, to discriminate amyloid-containing biopsies from CR-negative samples. Support Vector Machine algorithm was developed based on the quantitative readout (number of peptide spectrum matches) of each of the identified amyloid signature protein (Table 1) from the proteomics analysis of the biopsies from the training set. The test data set consisted of 22 amyloid-containing biopsies (“+”) and 23 corresponding controls without amyloid (“−”).

Signature Protein (s)	Correct/Total	Sensitivity	Specificity	PPV	NPV	Accuracy
ApoA4	+: 21/22 −: 22/23	0.95	0.96	0.95	0.96	0.96
ApoE	+: 22/22 −: 22/23	1.00	0.96	0.96	1.00	0.98
SAP	+: 21/22 −: 23/23	0.95	1.00	1.00	0.96	0.98
Clusterin	+: 20/22 −: 23/23	0.90	1.00	1.00	0.92	0.96
Vitronectin	+: 6/22 −: 23/23	0.27	1.00	1.00	0.59	0.64
Complement C9	+: 2/22 −: 23/23	0.09	1.00	1.00	0.53	0.56
Collagen alpha-1(VI) chain	+: 14/22 −: 21/23	0.64	0.91	0.88	0.72	0.78
Collagen alpha-2(VI) chain	+: 13/22 −: 20/23	0.59	0.87	0.81	0.69	0.73
Collagen alpha-3(VI) chain	+: 13/22 −: 20/23	0.59	0.87	0.81	0.69	0.73
Fibulin-1	+: 7/22 −: 23/23	0.32	1.00	1.00	0.61	0.67
ApoA4 and ApoE	+: 20/22 −: 22/23	0.91	0.96	0.95	0.92	0.93
ApoA4, ApoE, Clusterin	+: 22/22 −: 23/23	1.00	1.00	1.00	1.00	1.00
ApoA4, ApoE, Vitronectin	+: 22/22 −: 22/23	1.00	0.96	0.96	1.00	0.98
ApoA4, ApoE, Complement C9	+: 22/22 −: 22/23	1.00	0.96	0.96	1.00	0.98
ApoA4, ApoE, Collagen alpha−1(VI) chain	+: 20/22 −: 22/23	0.91	0.96	0.95	0.92	0.93
ApoA4, ApoE, Collagen alpha−2(VI) chain	+: 20/22 −: 23/23	0.91	1.00	1.00	0.92	0.96
ApoA4, ApoE, Collagen alpha−3(VI) chain	+: 21/22 −: 22/23	0.95	0.96	0.95	0.96	0.96
ApoA4, ApoE, Fibulin−1	+: 20/22 −: 23/23	0.91	1.00	1.00	0.92	0.96
ApoA4, ApoE and SAP	+: 22/22 −: 23/23	1.00	1.00	1.00	1.00	1.00

**Table 3:** Validation of the SVM-based models for recognizing tissue samples with amyloid deposits by testing on blinded validation data set consisting of 103 amyloid-containing tissue samples of different organ origin collected from amyloidosis patients.

Amyloid Signature Proteins	Correct/Total	Accuracy
ApoA4	99/103	0.96
ApoE	99/103	0.96
SAP	103/103	1.00
Clusterin	97/103	0.94
Vitronectin	64/103	0.62
Complement C9	26/103	0.25
Collagen alpha-1(VI) chain	83/103	0.81
Collagen alpha-2(VI) chain	82/103	0.80
Collagen alpha-3(VI) chain	84/103	0.82
Fibulin-1	72/103	0.70
ApoA4 and ApoE	100/103	0.97
ApoA4, ApoE, Clusterin	101/103	0.98
ApoA4, ApoE and Vitronectin	102/103	0.99
ApoA4, ApoE and Complement C9	102/103	0.99
ApoA4, ApoE and Collagen alpha-1(VI) chain	102/103	0.99
ApoA4, ApoE and Collagen alpha-2(VI) chain	102/103	0.99
ApoA4, ApoE and Collagen alpha-3(VI) chain	102/103	0.99
ApoA4, ApoE, and Fibulin-1	101/103	0.98
ApoA4, ApoE and SAP	103/103	1.00

**Table 4:** Subtype-specific proteins included in subtype classification model. These proteins are commonly observed in high levels in relation to their respective subtype and were, therefore, selected for the classifier.

#Accession	Protein Name	Subtype
P0DJI8	Serum amyloid A-1	AA
P0DJI9	Serum amyloid A-2	
P0DOX7	Immunoglobulin kappa light chain	AL-kappa
P01834	Immunoglobulin kappa constant	
P0DOX8	Immunoglobulin lambda-1 light chain	AL-lambda
P0DOY2	Immunoglobulin lambda constant 2	
P02766	Transthyretin	ATTR

**Table 5:** SVM-based classification of prospectively collected amyloid-containing samples. The validation set consisted of 103 Congo positive cases with confirmed diagnosis by IEM. In total, 69 ATTR cases, 21 AL-L cases and 4 AL-K was included in the validation set. For the 4-protein model, four ATTR-cases were misclassified as AL-L, whereas for the 7-protein model one ATTR sample was misclassified as AL-K.

Subtype Classification	Amyloidogenic Proteins	Correct/Total	Sensitivity	Specificity	PPV	NPV	Accuracy
AA	Serum amyloid A-1 protein (SA)	6/6	1.00	1.00	1.00	1.00	0.96 */0.99
	SA and Serum amyloid A-2 protein	6/6	1.00	1.00	1.00	1.00	
AL-K	Immunoglobulin kappa light chain (IgK)	4/4	1.00	1.00	1.00	1.00	
	IgK and Ig kappa constant	4/4	1.00	0.99	0.80	1.00	
AL-L	Ig lambda-1 light chain (IgL-1)	25/25	1.00	0.95	0.86	1.00	
	IgL-1 and Ig lambda constant 2	25/25	1.00	1.00	1.00	1.00	
ATTR	Transthyretin (four-protein model)	64/68	0.94	1.00	1.00	0.90	
	Transthyretin (seven-protein model)	67/68	0.99	1.00	1.00	0.97	

* Accuracy for the four protein model.

## Data Availability

The datasets generated and/or analyzed during the current study are not publicly available due to hospital guidelines and legislation regarding personal data. Data will be available from the corresponding author on reasonable request and with permission of Odense University Hospital Legal Department.

## Supplementary Materials

The following supporting information can be downloaded at: https://www.mdpi.com/article/10.3390/ijms23010319/s1.
