# Supplementary material for: Classification of Amyloidosis by Model-Assisted Mass Spectrometry-Based Proteomics
Source: Int J Mol Sci. 2021 Dec 28;23(1):319. doi: 10.3390/ijms23010319 (PMC8745254; doi:10.3390/ijms23010319)
Supplement: Supplementary file 1 [file ijms-23-00319-s001.zip › ijms-1512327-supplementary.pdf]

Supplementary Table S1. Number of samples included in study organized by tissue origins.

Organ/Tissue	Training	Test	Validation
	CR+/CR-	CR+/CR-	CR+
Bone	8/12	2/5	9
GIM	11/13	3/5	0
Heart	14/14	5/6	5
Kidney	8/10	6/6	6
Lung	6/4	4/	4
Others			22
Skin	6/2	2/1	10
Synovial			35
Tenosynovial			12
Total	53/55	22/23	103

Supplementary Table S3. Result from training data set of disease-state classification

Support Vector Machine algorithm was developed based on the quantitative readout (number of peptide spectrum matches) of each of the identified amyloid signature protein (Table 1) from the proteomics analysis of the biopsies from the training set. The training data set consisted of 53 amyloid-containing biopsies ("+") and 55 corresponding controls without amyloid ("-").

Signature protein	Correct/Total	Sensitivity	Specificity	PPV	NPV	Accuracy
ApoA4	+: 51/53<br>-: 52/55	0.96	0.95	0.94	0.96	0.95
ApoE	+: 45/53<br>-: 49/55	0.85	0.89	0.88	0.86	0.87
SAP	+: 50/53<br>-: 55/55	0.94	1.00	1.00	0.95	0.97
Clusterin	+: 52/53<br>-: 55/55	0.98	1.00	1.00	0.98	0.99
Vitronectin	+: 22/53<br>-: 55/55	0.42	1.00	1.00	0.64	0.71
Complement C9	+: 8/53<br>-: 55/55	0.15	1.00	1.00	0.55	0.58
Collagen alpha-1(VI) chain	+: 40/53<br>-: 50/55	0.75	0.91	0.89	0.79	0.83
Collagen alpha-2(VI) chain	+: 37/53<br>-: 48/55	0.70	0.87	0.84	0.75	0.79
Collagen alpha-3(VI) chain	+: 41/53<br>-: 46/55	0.77	0.84	0.82	0.79	0.81
Fibulin-1	+: 25/53<br>-: 55/55	0.47	1.00	1.00	0.66	0.74
ApoA4+ApoE	+: 52/53<br>-: 54/55	0.98	0.98	0.98	0.98	0.98
+ Clusterin	+: 53/53<br>-: 55/55	1.00	1.00	1.00	1.00	1.00
+ Vitronectin	+: 52/53<br>-: 54/55	0.98	0.98	0.98	0.98	0.98
+ Complement C9	+: 53/53<br>-: 54/55	1.00	0.98	0.98	1.00	0.99
+ Collagen alpha-1(VI) chain	+: 52/53<br>-: 54/55	0.98	0.98	0.98	0.98	0.98

+ Collagen alpha-2(VI) chain	+: 53/53<br>-: 55/55	1.00	1.00	1.00	1.00	1.00
+ Collagen alpha-3(VI) chain	+: 52/53<br>-: 54/55	0.98	0.98	0.98	0.98	0.98
+ Fibulin-1	+: 53/53<br>-: 55/55	1.00	1.00	1.00	1.00	1.00
ApoA4+ApoE+SAP	+: 53/53<br>-: 55/55	1.00	1.00	1.00	1.00	1.00
